# Supplementary material for: The interactome of intact mitochondria by cross-linking mass spectrometry provides evidence for coexisting respiratory supercomplexes
Source: Mol Cell Proteomics. 2017 Dec 8;17(2):216–32. doi: 10.1074/mcp.RA117.000470 (PMC5795388; doi:10.1074/mcp.RA117.000470)
Supplement: Supplemental Data [file supp_RA117.000470_133922_0_supp_23978_fzffwf.zip › spectra_annotation/mito_spectra_annotation/424-1-3-1-9-1.pdf]

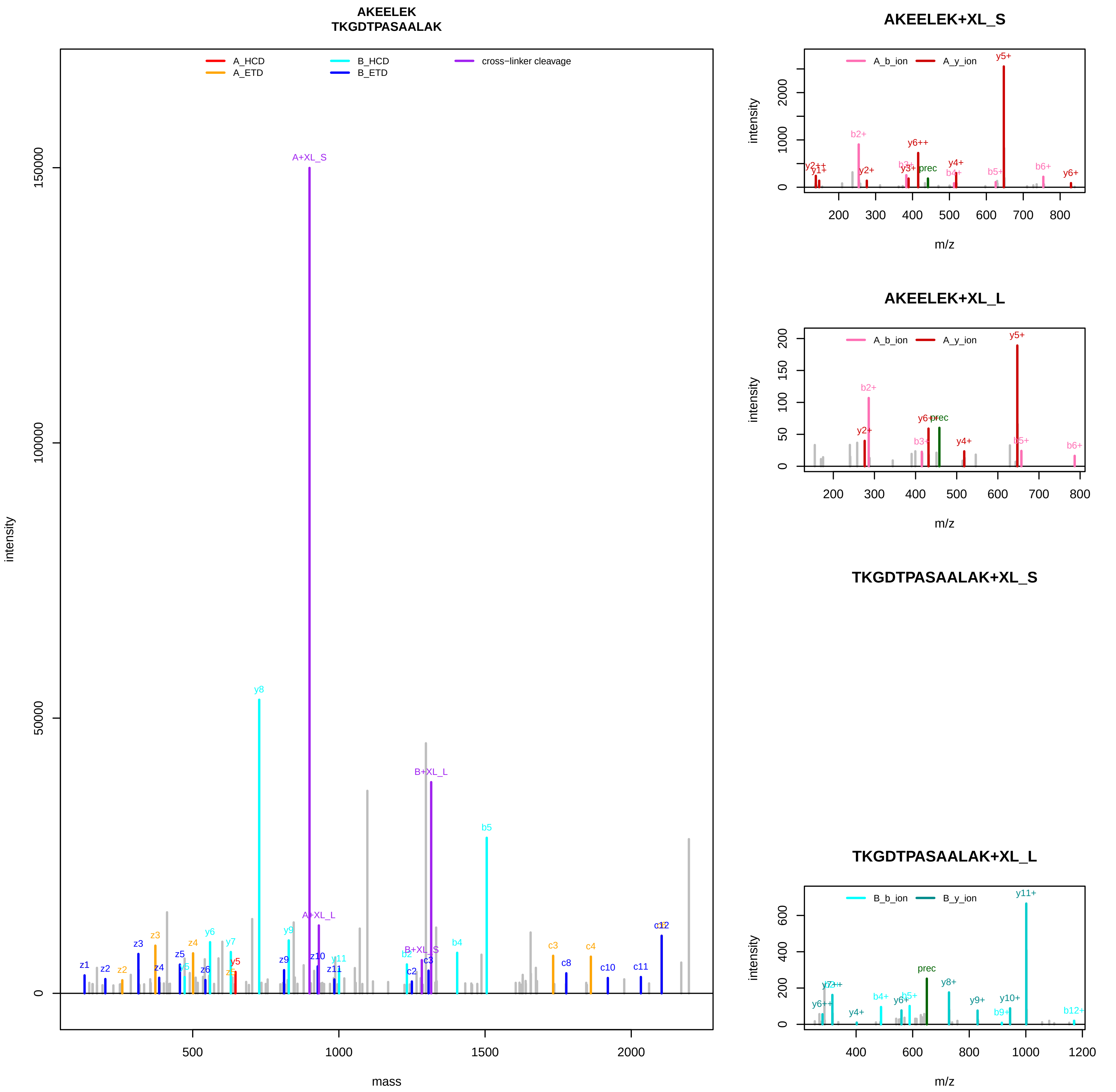
AKEELEK
TKGDTPASAALAK
A_HCD
A_ETD
B_HCD
B_ETD
cross−linker cleavage
A+XL_S
A+XL_L
B+XL_L
B+XL_S
intensity
mass
0
50000
100000
150000
500
1000
1500
2000
y8
y9
y6
y7
y5
y11
b2
b4
b5
z1
z2
z3
z4
z5
z6
z9
z10
z11
c2
c3
c8
c10
c11
c12
z2
z3
z4
z5
c3
c4
AKEELEK+XL_S
A_b_ion
A_y_ion
y5+
b2+
y6++
y2++
y1+
y2+
y3+
prec
y4+
b4+
b5+
b6+
y6+
0
1000
2000
200
300
400
500
600
700
800
m/z
AKEELEK+XL_L
y5+
b2+
y2+
y6+
prec
b3+
y4+
b5+
b6+
0
50
100
150
200
TKGDTPASAALAK+XL_S
TKGDTPASAALAK+XL_L
B_b_ion
B_y_ion
y11+
prec
y8+
y9+
y10+
b9+
b12+
y6++
y4+
b4+
y6+
b5+
0
200
400
600
1000
1200
